# Supplementary material for: Association Between Healthy Dietary Patterns and Chronic Kidney Disease in Patients with Diabetes: Findings from Korean National Health and Nutrition Examination Survey 2019–2021
Source: Nutrients. 2025 May 7;17(9):1600. doi: 10.3390/nu17091600 (PMC12073184; doi:10.3390/nu17091600)
Supplement: Supplementary file 1 [file nutrients-17-01600-s001.zip › Supp_DM_KHEI_CKD_KNHANES_250409_Nutrients.pdf]

Supplemental Material

Association between Healthy Dietary Patterns and Chronic Kidney Disease in Patients with Diabetes: Findings from the Korean National Health and Nutrition Examination Survey 2019-2021

M Kim et al.

Table of contents

Table S1. Korean Healthy Eating Index components and the standards for scoring

Table S2. Subgroup analysis of the prevalence of chronic kidney disease using the Korean Healthy Eating Index as a continuous variable

Table S3. Comparison of the Korean Healthy Eating Index components according to the presence of chronic kidney disease

Table S4. Subgroup analysis of comparison of the Korean Healthy Eating Index components according to the presence of chronic kidney disease

Table S1. Korean Healthy Eating Index components and the standards for scoring.

Category	Component	Score range	Standard for maximum score	Standard for minimum score
**Adequacy**	Have breakfast	0–10	5–7 times/week	0 times/week
	Mixed grains intake	0–5	≥ 0.3 serving/day	0 serving/day
	Total fruits intake	0–5	• Men aged 19–64 years: ≥ 3 serving/day<br>• Men aged 65 years and overs: ≥ 2 serving/day<br>• Women aged 19–64 years: ≥ 2 serving/day<br>• Women aged 65 years and overs: ≥ 1 serving/day	0 serving/day
	Fresh fruits intake	0–5	• Men aged 19–64 years: ≥ 1.5 serving/day<br>• Women aged 19–64 years: ≥ 1 serving/day<br>• Men aged 65 years and overs: ≥ 1 serving/day<br>• Women aged 65 years and over: ≥ 0.5 serving/day	0 serving/day
	Total vegetables intake	0–5	• Men and women aged 19–64 years: ≥ 8 serving/day<br>• Men aged 65 years and overs: ≥ 8 serving/day<br>• Women aged 65 years and overs: ≥ 6 serving/day	0 serving/day
	Vegetables intake excluding Kimchi and pickled vegetables intake	0–5	• Men and women aged 19–64 years: ≥ 5 serving/day<br>• Men aged 65 years and overs: ≥ 5 serving/day<br>• Women aged 65 years and overs: ≥ 3 serving/day	0 serving/day
	Meat, fish, eggs, and beans intake	0–10	• Men aged 19–64 years: ≥ 5 serving/day<br>• Women aged 19–64 years: ≥ 4 serving/day<br>• Men aged 65 years and overs: ≥ 4 serving/day<br>• Women aged 65 years and overs: ≥ 2.5 serving/day	0 serving/day
	Milk and dairy products intake	0–10	≥ 1 serving/day	0 serving/day
**Moderation**	Percentage of energy from saturated fatty acid	0–10	< 7% of total energy intake	> 10% of total energy intake
	Sodium intake	0–10	≤ 2,000 mg/day	> 6,500 mg/day
	Percentage of energy from sweets and beverages	0–10	≤ 10% of total energy intake	> 20% of total energy intake
**Balance**	Percentage of energy from carbohydrate	0–5	55–65% of total energy intake	< 50% or > 75% of total energy intake
	Percentage of energy intake from fat	0–5	15–30% of total energy intake	< 10% or > 35% of total energy intake
	Energy intake	0–5	75–125% of the EER	< 60% or > 140% of EER

EER, estimated energy requirement; KDRI, Dietary Reference Intakes for Koreans; KHEI, Korean Healthy Eating Index. The frequency of each food intake (serving) that was used to determine the KHEI was calculated based on one serving size proposed by the 2015 KDRI: Frequency of Intake (Serving) = Amount of Intake (g)/One Serving Size (g).

Table S2. Subgroup analysis of the prevalence of chronic kidney disease using the Korean Healthy Eating Index as a continuous variable

	Subgroup	N	Event	%	aOR (95% CI)	P-value	P for interaction
**Age**	<65 years	926	188	45.4	0.982(0.967,0.997)	0.02	0.29
	≥65 years	1065	365	54.6	0.987(0.974,1.000)	0.05	
**Sex**	Men	1000	283	55.1	0.986(0.972,1.000)	0.06	0.43
	Women	991	270	44.9	0.984(0.974,0.994)	0.006	
**Hypertension**	No	770	140	27.5	0.975(0.958,0.992)	0.005	0.02
	Yes	1221	413	72.5	0.989(0.976,1.002)	0.09	
**Income status**	<20%	492	194	32.7	0.980(0.962,0.999)	0.04	0.68
	≥20%	1499	359	67.3	0.985(0.973,0.996)	0.01	
**Education level**	≤Middle school	1039	340	53.3	0.990(0.977,1.003)	0.14	0.34
	≥High school	952	213	46.8	0.979(0.965,0.994)	0.006	
**Number of households members**	1-person	397	139	76	1.002(0.983,1.020)	0.87	0.33
	≥2-persons	1594	414	24	0.979(0.968,0.990)	<0.001	
**Residence area**	Urban	828	224	43.4	0.997(0.982,1.012)	0.72	0.04
	Rural	1163	329	56.6	0.974(0.962,0.987)	<0.001	
**Occupation**	No	988	332	55.7	0.986(0.973,0.998)	0.03	0.76
	Yes	1003	221	44.3	0.981(0.966,0.996)	0.02	
**Marriage**	No	92	29	7.8	0.999(0.956,1.043)	0.96	0.49
	Yes	1899	524	92.2	0.982(0.972,0.992)	<0.001	

The odds ratio of prevalent CKD was calculated according to the KHEI as a continuous variable. SE, standard error; aOR, adjusted odds ratio; CI, confidence interval. aOR was adjusted for age, sex, BMI, history of hypertension and dyslipidemia, education level, income status, occupation status, smoking, alcohol consumption, and physical activity.

Table S3. Comparison of the Korean Healthy Eating Index components according to the presence of chronic kidney disease.

KHEI (Mean ± SE)		CKD		P-value
		No	Yes	
**Total score**				
Unadjusted		63.30±0.38	61.91±0.64	0.06
Age, sex adjusted		64.63±0.36	61.74±0.59	<0.001
Model 1[a]		64.63±0.36	61.80±0.60	<0.001
Model 2[b]		62.96±0.51	60.64±0.68	0.002
**Category**	**Components**			
**Adequacy**	**Have breakfast**			
	Unadjusted	8.29±0.11	8.29±0.19	1.00
	Age, sex adjusted	8.72±0.10	8.20±0.17	0.013
	Model 1	8.70±0.10	8.20±0.17	0.016
	Model 2	8.43±0.16	8.00±0.20	0.040
	**Mixed grains intake**			
	Unadjusted	2.48±0.07	2.43±0.12	0.72
	Age, sex adjusted	2.65±0.07	2.41±0.11	0.06
	Model 1	2.64±0.07	2.42±0.12	0.10
	Model 2	2.51±0.09	2.33±0.12	0.16
	**Total fruits intake**			
	Unadjusted	2.20±0.07	2.00±0.11	0.14
	Age, sex adjusted	2.40±0.07	2.00±0.11	0.002
	Model 1	2.39±0.07	2.03±0.11	0.006
	Model 2	2.11±0.08	1.82±0.12	0.025
	**Fresh fruits intake**			
	Unadjusted	2.40±0.08	2.17±0.12	0.09
	Age, sex adjusted	2.58±0.07	2.18±0.12	0.003
	Model 1	2.57±0.08	2.22±0.12	0.012
	Model 2	2.25±0.09	1.96±0.13	0.038
	**Total vegetables intake**			
	Unadjusted	3.78±0.04	3.67±0.08	0.21
	Age, sex adjusted	3.81±0.04	3.64±0.07	0.049
	Model 1	3.81±0.04	3.63±0.08	0.030
	Model 2	3.80±0.05	3.68±0.08	0.15
	**Vegetables intake excluding Kimchi and pickled vegetables**			
	Unadjusted	3.46±0.05	3.24±0.08	0.019
	Age, sex adjusted	3.48±0.05	3.23±0.08	0.008
	Model 1	3.48±0.05	3.23±0.08	0.008
	Model 2	3.50±0.06	3.32±0.09	0.07
	**Meat, fish, eggs, and beans intake**			
	Unadjusted	7.15±0.10	6.58±0.17	0.002
	Age, sex adjusted	7.08±0.10	6.57±0.17	0.008
	Model 1	7.11±0.10	6.59±0.17	0.006
	Model 2	7.30±0.12	6.97±0.18	0.08
	**Milk and dairy products intake**			
	Unadjusted	2.79±0.14	2.2±0.20	0.014
	Age, sex adjusted	2.79±0.13	2.24±0.20	0.022
	Model 1	2.83±0.13	2.21±0.20	0.013
	Model 2	2.43±0.16	1.86±0.22	0.020
**Moderation**	**Energy from saturated fatty acid**			
	Unadjusted	7.85±0.11	8.44±0.20	0.001
	Age, sex adjusted	8.09±0.10	8.41±0.20	0.16

	Model 1	8.07±0.11	8.40±0.20	0.16
	Model 2	7.73±0.16	8.00±0.23	0.24
	**Sodium intake**			
	Unadjusted	6.79±0.11	7.22±0.15	0.019
	Age, sex adjusted	7.06±0.09	7.27±0.14	0.22
	Model 1	7.03±0.10	7.28±0.15	0.16
	Model 2	6.78±0.13	6.90±0.16	0.49
	**Energy from sweets and beverages**			
	Unadjusted	7.01±0.11	7.28±0.20	0.23
	Age, sex adjusted	6.95±0.11	7.24±0.20	0.22
	Model 1	6.96±0.11	7.20±0.21	0.32
	Model 2	7.27±0.13	7.48±0.22	0.38
**Balance**	**Energy from carbohydrate**			
	Unadjusted	2.56±0.07	2.32±0.10	0.048
	Age, sex adjusted	2.47±0.07	2.31±0.10	0.19
	Model 1	2.47±0.07	2.33±0.10	0.26
	Model 2	2.43±0.09	2.31±0.12	0.33
	**Energy from fat**			
	Unadjusted	3.47±0.07	3.17±0.11	0.015
	Age, sex adjusted	3.39±0.07	3.17±0.11	0.08
	Model 1	3.39±0.07	3.19±0.11	0.11
	Model 2	3.34±0.09	3.20±0.12	0.27
	**Energy intake**			
	Unadjusted	3.08±0.07	2.90±0.11	0.17
	Age, sex adjusted	3.16±0.07	2.88±0.11	0.040
	Model 1	3.17±0.07	2.89±0.11	0.040
	Model 2	3.07±0.09	2.83±0.13	0.08

[a]Multivariable model 1was adjusted for age, sex, body mass index (BMI), and history of hypertension.
[b]Multivariable model 2 was adjusted for age, sex, BMI, history of hypertension and dyslipidemia, education level, income status, occupation status, smoking, alcohol consumption, and physical activity.
KHEI, Korean healthy eating index; CKD, Chronic kidney disease. CKD is defined as eGFR<60mL/min/1.73m^2 or UACR≥30mg/g.

Table S4. Subgroup analysis of comparison of the Korean Healthy Eating Index components according to the presence of chronic kidney disease.

		CKD	Age		Sex		HTN		Income status		Education level		Household members		Residence area		Occupation		Marriage	
			<65	≥65	Men	Women	No	Yes	<20%	≥20%	≤Middle	≥High	1-person	≥2-persons	Urban	Rural	No	Yes	No	Yes
KHEI (Total)		No	60.7±0.6	64.5±0.8	63.4±0.6	61.4±1.3	61.6±0.7	64.0±0.7	62.1±1.4	63.3±0.5	64.1±0.9	62.3±0.6	61.3±1.6	63.4±0.5	63.2±0.8	63.0±0.7	62.6±0.9	62.9±0.6	56.9±2.4	63.3±0.5
		Yes	58.2±1.0	63.1±0.1	61.5±0.9	58.6±1.5	57.9±1.2	62.4±0.9	59.2±1.4	61.1±0.8	62.6±1.0	59.5±1.0	61.6±1.4	60.6±0.8	62.8±1.0	59.2±0.9	60.4±1.1	60.4±0.9	57.3±2.3	60.7±0.7
		P	**0.020**	0.05	**0.049**	**0.006**	**0.004**	0.10	**0.046**	**0.011**	0.13	**0.008**	0.84	**0.001**	0.71	**<0.001**	**0.036**	**0.013**	0.91	**0.001**
Category	Components																			
Adequacy	Have breakfast	No	7.8±0.2	8.8±0.2	8.7±0.2	7.3±0.6	8.1±0.3	8.6±0.2	8.4±0.4	8.4±0.2	8.8±0.2	8.0±0.2	7.8±0.3	8.5±0.2	8.6±0.2	8.4±0.2	8.5±0.3	8.3±0.2	6.0±0.6	8.6±0.2
		Yes	6.9±0.3	9.0±0.2	7.9±0.3	7.3±0.6	7.6±0.4	8.3±0.2	8.0±0.4	7.9±0.2	8.6±0.2	7.3±0.3	7.6±0.3	8.2±0.2	7.9±0.3	8.2±0.3	8.2±0.3	7.8±0.3	6.3±0.7	8.2±0.2
		P	**0.015**	0.24	**0.009**	0.94	0.15	0.19	0.21	0.08	0.48	0.07	0.56	0.12	0.07	0.41	0.26	0.07	0.69	0.08
	Mixed grains intake	No	2.2±0.1	2.8±0.2	2.5±0.1	2.8±0.3	2.3±0.1	2.7±0.1	2.7±0.3	2.5±0.1	2.8±0.2	2.3±0.1	2.7±0.3	2.5±0.1	2.6±0.1	2.4±0.1	2.5±0.2	2.4±0.1	2.5±0.4	2.5±0.1
		Yes	2.0±0.2	2.7±0.2	2.3±0.2	2.6±0.3	2.0±0.3	2.6±0.2	2.5±0.3	2.3±0.1	2.6±0.2	2.2±0.2	2.5±0.3	2.3±0.1	2.6±0.2	2.2±0.2	2.3±0.2	2.3±0.2	1.5±0.5	2.4±0.1
		P	0.22	0.46	0.32	0.30	0.30	0.58	0.46	0.23	0.19	0.48	0.40	0.17	0.67	0.12	0.14	0.62	0.13	0.27
	Total fruits Intake	No	1.8±0.1	2.6±0.2	1.9±0.1	1.8±0.2	2.1±0.1	2.1±0.1	1.9±0.2	2.2±0.1	2.3±0.2	2.1±0.1	1.7±0.2	2.2±0.1	2.3±0.1	2.0±0.1	2.3±0.1	2.0±0.1	1.4±0.3	2.2±0.1
		Yes	1.6±0.2	2.2±0.2	1.5±0.2	1.7±0.2	1.4±0.2	2.1±0.2	1.6±0.2	2.0±0.2	2.0±0.2	1.8±0.2	1.8±0.2	1.9±0.1	2.0±0.2	1.6±0.2	2.3±0.2	1.5±0.2	1.4±0.4	1.8±0.1
		P	0.50	**0.009**	**0.020**	0.56	**0.001**	0.62	0.21	0.11	0.07	0.25	0.93	**0.027**	0.30	**0.032**	0.92	**0.002**	0.97	**0.018**
	Fresh fruits intake	No	2.0±0.1	2.7±0.2	2.1±0.1	2.0±0.3	2.3±0.1	2.3±0.1	2.0±0.2	2.4±0.1	2.5±0.2	2.2±0.1	1.8±0.2	2.4±0.1	2.4±0.1	2.2±0.1	2.3±0.2	2.2±0.1	1.5±0.3	2.3±0.1
		Yes	1.8±0.2	2.3±0.2	1.7±0.2	1.9±0.3	1.6±0.2	2.2±0.2	1.8±0.2	2.1±0.2	2.2±0.2	2.0±0.2	2.0±0.2	2.0±0.2	2.3±0.2	1.7±0.2	2.4±0.2	1.6±0.2	1.6±0.5	2.0±0.1
		P	0.38	**0.040**	**0.047**	0.49	**0.003**	0.59	0.41	0.09	0.11	0.28	0.32	**0.019**	0.64	**0.017**	0.63	**0.002**	0.81	**0.027**
	Total vegetables intake	No	3.7±0.1	3.8±0.1	3.9±0.1	3.5±0.3	3.7±0.1	3.8±0.1	3.8±0.2	3.8±0.1	3.8±0.1	3.8±0.1	3.8±0.1	3.8±0.1	3.8±0.1	3.8±0.1	3.7±0.1	3.9±0.1	3.2±0.3	3.8±0.1
		Yes	3.6±0.1	3.8±0.1	3.9±0.1	3.3±0.3	3.6±0.2	3.8±0.1	3.5±0.2	3.8±0.1	3.6±0.1	3.7±0.1	3.7±0.2	3.7±0.1	3.8±0.1	3.5±0.1	3.5±0.1	3.9±0.1	3.4±0.2	3.7±0.1
		P	0.31	0.52	0.67	0.14	0.30	0.43	0.12	0.51	0.16	0.49	0.44	0.34	0.75	**0.024**	0.09	0.88	0.59	0.10
	Vegetables intake excluding Kimchi and pickled vegetables	No	3.4±0.1	3.5±0.1	3.4±0.1	3.3±0.3	3.5±0.1	3.5±0.1	3.5±0.2	3.5±0.1	3.4±0.1	3.6±0.1	3.5±0.2	3.5±0.1	3.5±0.1	3.5±0.1	3.4±0.1	3.6±0.1	2.9±0.3	3.5±0.1
		Yes	3.3±0.1	3.4±0.1	3.4±0.1	3.0±0.3	3.2±0.2	3.4±0.1	3.0±0.2	3.4±0.1	3.3±0.1	3.4±0.1	3.5±0.2	3.3±0.1	3.5±0.1	3.2±0.1	3.2±0.2	3.5±0.1	3.2±0.4	3.3±0.1
		P	0.22	0.31	0.66	0.05	0.18	0.25	**0.024**	0.47	0.40	0.08	0.78	0.09	0.95	**0.022**	0.05	0.57	0.54	**0.039**
	Meat, fish, Eggs, and beans intake	No	7.4±0.2	7.0±0.2	7.3±0.1	6.9±0.5	7.2±0.2	7.4±0.2	6.8±0.3	7.5±0.1	6.9±0.2	7.7±0.1	6.5±0.4	7.4±0.1	7.4±0.2	7.2±0.2	6.9±0.2	7.6±0.1	7.1±0.5	7.3±0.1
		Yes	7.1±0.3	6.6±0.3	7.0±0.2	6.5±0.5	7.0±0.3	6.9±0.2	6.2±0.4	7.2±0.2	6.6±0.3	7.4±0.3	6.9±0.4	7.0±0.2	7.5±0.3	6.6±0.2	6.7±0.3	7.1±0.3	6.8±0.5	6.9±0.2
		P	0.34	0.11	0.28	0.20	0.64	0.05	0.20	0.17	0.26	0.27	0.34	**0.030**	0.75	**0.023**	0.42	0.07	0.71	0.06
	Milk and dairy	No	2.5±0.2	2.6±0.3	2.7±0.2	1.8±0.4	2.1±0.2	2.7±0.2	2.9±0.4	2.5±0.2	2.3±0.3	2.9±0.2	2.7±0.5	2.4±0.2	2.3±0.2	2.5±0.2	2.6±0.3	2.4±0.2	3.0±0.8	2.5±0.2

	**products intake**	**Yes**	2.0±0.4	1.9±0.3	1.8±0.3	1.5±0.4	2.0±0.4	1.9±0.3	2.0±0.4	1.9±0.3	2.1±0.3	1.9±0.3	1.6±0.5	1.9±0.3	1.7±0.4	1.9±0.3	1.8±0.3	1.9±0.3	2.0±0.9	1.9±0.2
		**P**	0.16	**0.049**	**0.018**	0.33	0.87	**0.007**	**0.027**	0.08	0.42	**0.018**	0.06	0.07	0.13	0.08	**0.013**	0.25	0.42	**0.012**
**Moderation**	**Energy from saturated fatty acid**	**No**	7.3±0.2	8.1±0.3	8.0±0.2	8.2±0.4	7.7±0.2	7.8±0.2	7.7±0.4	7.7±0.2	8.3±0.2	7.1±0.2	8.1±0.3	7.7±0.2	7.5±0.2	7.9±0.2	7.7±0.3	7.7±0.2	7.3±0.5	7.7±0.2
		**Yes**	7.7±0.4	8.3±0.3	8.6±0.2	8.0±0.5	7.7±0.5	8.1±0.3	7.7±0.4	8.0±0.3	8.3±0.3	7.7±0.4	8.5±0.5	7.9±0.3	8.2±0.4	7.9±0.3	7.7±0.4	8.3±0.3	7.8±0.8	8.0±0.2
		**P**	0.31	0.39	**0.028**	0.52	0.87	0.15	0.96	0.16	0.81	0.18	0.36	0.33	**0.043**	0.75	1.00	0.05	0.57	0.26
	**Sodium intake**	**No**	6.2±0.2	7.4±0.2	6.1±0.2	8.1±0.3	6.8±0.2	6.8±0.2	7.0±0.3	6.6±0.1	7.1±0.2	6.3±0.2	6.8±0.3	6.7±0.1	6.7±0.2	6.9±0.2	7.2±0.2	6.3±0.2	5.7±0.6	6.8±0.1
		**Yes**	6.4±0.3	7.4±0.2	6.3±0.2	8.0±0.4	6.7±0.3	7.0±0.2	7.3±0.3	6.7±0.2	7.4±0.3	6.2±0.3	7.1±0.3	6.8±0.2	6.7±0.3	7.1±0.2	7.2±0.3	6.6±0.3	6.1±0.8	7.0±0.2
		**P**	0.54	0.98	0.37	0.65	0.81	0.34	0.46	0.68	0.17	0.69	0.29	0.87	0.92	0.36	0.90	0.37	0.65	0.47
	**Energy from sweets and beverages**	**No**	7.3±0.2	6.9±0.2	7.4±0.2	7.8±0.4	7.1±0.2	7.4±0.2	7.7±0.3	7.1±0.1	7.2±0.2	7.1±0.2	7.4±0.3	7.2±0.1	7.3±0.2	7.3±0.2	7.0±0.3	7.4±0.1	7.1±0.6	7.2±0.1
		**Yes**	7.4±0.3	7.2±0.3	7.8±0.3	7.7±0.4	7.2±0.4	7.6±0.2	8.3±0.4	7.2±0.3	7.7±0.3	7.0±0.3	7.6±0.4	7.5±0.3	7.5±0.3	7.5±0.3	7.0±0.4	7.8±0.3	8.0±0.6	7.4±0.2
		**P**	0.78	0.32	0.14	0.86	0.68	0.34	0.14	0.74	0.12	0.88	0.60	0.40	0.53	0.58	0.98	0.25	0.27	0.45
**Balance**	**Energy from carbohydrate**	**No**	2.6±0.1	2.2±0.2	2.7±0.1	2.1±0.3	2.4±0.1	2.4±0.1	2.1±0.2	2.6±0.1	2.3±0.2	2.7±0.1	2.2±0.2	2.5±0.1	2.5±0.1	2.4±0.1	2.4±0.1	2.5±0.1	2.8±0.4	2.4±0.1
		**Yes**	2.4±0.2	2.1±0.2	2.7±0.2	1.8±0.3	2.2±0.2	2.4±0.1	2.1±0.3	2.4±0.1	2.2±0.2	2.6±0.2	2.4±0.3	2.3±0.1	2.6±0.2	2.1±0.2	2.2±0.2	2.4±0.2	2.8±0.4	2.2±0.1
		**P**	0.46	0.39	0.91	0.20	0.40	0.61	0.84	0.22	0.69	0.52	0.61	0.13	0.60	0.11	0.30	0.51	0.90	0.17
	**Energy from fat**	**No**	3.5±0.1	3.2±0.1	3.6±0.1	3.0±0.3	3.4±0.1	3.3±0.1	2.7±0.3	3.5±0.1	3.2±0.2	3.6±0.1	3.1±0.2	3.4±0.1	3.4±0.1	3.3±0.1	3.3±0.2	3.4±0.1	4.0±0.3	3.3±0.1
		**Yes**	3.4±0.2	3.1±0.2	3.4±0.2	3.0±0.3	3.1±0.2	3.3±0.1	2.6±0.3	3.4±0.2	3.1±0.1	3.5±0.2	3.1±0.2	3.2±0.1	3.5±0.2	3.0±0.2	3.2±0.2	3.2±0.2	3.1±0.4	3.2±0.1
		**P**	0.38	0.56	0.14	0.94	0.18	0.72	0.57	0.21	0.62	0.45	0.91	0.17	0.64	0.07	0.61	0.23	**0.039**	0.36
	**Energy intake**	**No**	2.9±0.1	3.4±0.2	3.1±0.1	2.8±0.3	3.0±0.1	3.2±0.1	2.9±0.2	3.1±0.1	3.2±0.2	3.1±0.1	3.0±0.2	3.1±0.1	2.9±0.1	3.2±0.1	3.0±0.2	3.2±0.1	2.5±0.4	3.1±0.1
		**Yes**	2.7±0.2	3.1±0.2	3.1±0.2	2.3±0.4	2.6±0.3	3.0±0.2	2.6±0.3	2.9±0.2	2.8±0.2	3.0±0.2	3.3±0.3	2.7±0.2	2.9±0.2	2.8±0.2	2.9±0.2	2.7±0.2	3.3±0.5	2.8±0.1
		**P**	0.27	0.20	0.89	**0.009**	0.12	0.33	0.25	0.16	**0.037**	0.57	0.40	**0.017**	0.87	**0.014**	0.81	**0.032**	0.28	**0.032**

The KHEI and each category of KHEI are presented as adjusted mean ± standard error (SE). Age, sex, body mass index, history of hypertension and dyslipidemia, education level, income status, occupation status, smoking, alcohol consumption, and physical activity were adjusted. KHEI, Korean healthy eating index; CKD, Chronic kidney disease. CKD is defined as eGFR<60mL/min/1.73m^2 or UACR≥30mg/g.
